# Directional induction of neural stem cells, a new therapy for neurodegenerative diseases and ischemic stroke

**DOI:** 10.1038/s41420-023-01532-9

**Published:** 2023-07-01

**Authors:** Luwei Nie, Dabao Yao, Shiling Chen, Jingyi Wang, Chao Pan, Dongcheng Wu, Na Liu, Zhouping Tang

**Affiliations:** 1 Department of Neurology, Tongji Hospital, Tongji Medical College, Huazhong University of Science and Technology, Wuhan, 430030 Hubei China; 2 Department of Biochemistry and Molecular Biology, Wuhan University School of Basic Medical Sciences, Wuhan, 430030 China; 3 Wuhan Hamilton Biotechnology Co., Ltd., Wuhan, 430030 China

**Keywords:** Neuroscience, Neural stem cells

## Abstract

Due to the limited capacity of the adult mammalian brain to self-repair and regenerate, neurological diseases, especially neurodegenerative disorders and stroke, characterized by irreversible cellular damage are often considered as refractory diseases. Neural stem cells (NSCs) play a unique role in the treatment of neurological diseases for their abilities to self-renew and form different neural lineage cells, such as neurons and glial cells. With the increasing understanding of neurodevelopment and advances in stem cell technology, NSCs can be obtained from different sources and directed to differentiate into a specific neural lineage cell phenotype purposefully, making it possible to replace specific cells lost in some neurological diseases, which provides new approaches to treat neurodegenerative diseases as well as stroke. In this review, we outline the advances in generating several neuronal lineage subtypes from different sources of NSCs. We further summarize the therapeutic effects and possible therapeutic mechanisms of these fated specific NSCs in neurological disease models, with special emphasis on Parkinson’s disease and ischemic stroke. Finally, from the perspective of clinical translation, we compare the strengths and weaknesses of different sources of NSCs and different methods of directed differentiation, and propose future research directions for directed differentiation of NSCs in regenerative medicine.

## Facts

- Neural stem cells from different sources can be induced to differentiate into mature and functional neurons or glial cells in vitro.
- Transplantation of pre-differentiated neural stem cells can differentiate and mature into a specific type of cells, promoting the recovery of neurodegenerative disease or stroke models.
- Currently, dopaminergic neurons derived from human embryonic stem cells that undergo the neural stem cells stage are being tested in clinical trials in patients with Parkinson’s disease.

## Open questions

- For a neurodegenerative disease or stroke, which source of neural stem cells and which directed differentiation method will enable the transplanted cells to meet good manufacturing practices guideline?
- What is the optimal time window of the differentiation of neural stem cells for transplantation?
- What is the underlying mechanism of cell replacement in transplanted predifferentiated neural stem cells?

## Introduction

Neurodegenerative diseases (NDs) are a heterogeneous group of disorders that characterized by progressive and selective losses of neurons [1, 2], resulting in loss of sensation, movement and memory impairment, which are represented by Parkinson’s disease (PD), Huntington’s disease (HD), amyotrophic lateral sclerosis (ALS) and multiple sclerosis (MS) [3]. Ischemic stroke, the most common type of stroke, causes neuronal and non-neuronal death in the ischemic core due to decreased blood flow to part of the brain. Given enough time, reversible loss of tissue function in the ischemic penumbra can be permanent [4, 5]. These diseases directly threaten the lives of patients and bring a heavy economic burden to family and society [6]. However, current treatments involved in these diseases are not curative and relatively limited, most of which can relieve symptoms and delay the course of diseases [7, 8].

Nerve repair and regeneration therapy is an ideal way to treat neurological diseases. A great deal of work has been done in this area, mainly from both endogenous and exogenous aspects to promote nerve repair and regeneration [9]. Neural stem cells (NSCs) are a class of multipotent cells defined on the basis of their robust self-renewal capacity and ability to differentiate into various central nervous system (CNS) neuronal and glial cell types [10, 11]. Endogenous neurogenesis mediated by NSCs has been shown in several pathological conditions, such as epilepsy, MS, ischemic stroke, and AD [12], but endogenous repair alone is insufficient. NSCs transplantation strategy, as a type of regenerative medicine, has attracted increasing attention in the treatment of NDs [13]. Moreover, as the field of stem cells advances, the source of NSCs for transplantation has expanded from direct isolation of brain tissue initially to differentiation from pluripotent stem cells (PSCs) and transdifferentiation of somatic cells. So far, NSC-based therapies have been implemented in many rodent models of NDs and ischemic stroke, and several studies proposed potential mechanisms to explain the disease-improving effects of NSCs, including neuroinflammation inhibition, neuronal replacement, immunomodulation and neurotrophic support, which promotes the recovery of ND and stroke models [14–18]. Now clinical trials exploring the feasibility of NSCs treatment for neurological diseases are being conducted. Most studies based on NSCs therapy involve direct transplantation of NSCs from different sources into animal disease models.

However, non-negligible challenges of the directly transplanted NSCs are the low survival and irrational differentiation [19–22]. In both NDs and ischemic stroke, chronic or acute activation of innate immune cells in the CNS can be observed [23, 24]. The host micro-environment induced by a neuro-inflammatory response may play a critical role in the survival and differentiation of transplanted NSCs [25–27]. In addition, autophagy, which is involved in inflammatory pathways, has been demonstrated to regulate the differentiation of transplanted NSCs [28]. In animal models of spinal cord injury, transplanted NSCs were influenced by the neurotoxic inflammatory microenvironment and most of them differentiated into astrocytes, resulting in further aggravation [29]. Thus, the inflammatory response may adversely affect the ability of transplanted NSCs to participate in functional recovery. Further, the pathology of NDs is characterized by the selective loss of specific neurons or glial cells in restricted brain regions [30], such as midbrain dopaminergic (DAergic) neuron death in PD, medium spiny γ-aminobutyric acid–mediated (GABAergic) neurons (MSNs) loss in HD, degeneration of cholinergic motor neurons in ALS, and oligodendrocytes loss in MS. Compared with direct transplantation of NSCs, induced differentiation into specific phenotypes may be more amenable to replace lost cells in the CNS.

**Fig. 1:**
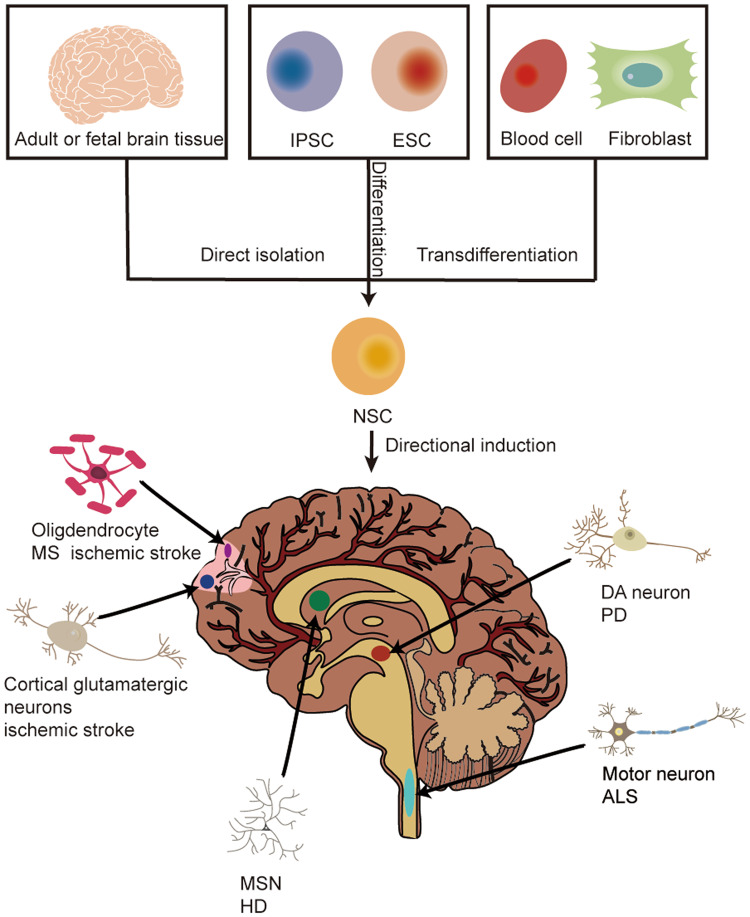
The directional differentiation of neural stem cells from different sources. Currently, NSCs can be obtained from three ways: isolate from primary CNS tissues, mainly including adult and fetal brain tissue; differentiate from pluripotent stem cells, including iPSCs and ESCs; transdifferentiate from somatic cells, such as blood cells and fibroblasts. NSCs derived from these three sources can be further processed in vitro to control their fate after transplantation in NDs models, thus replacing the lost cells. In NDs, NSCs can be induced to differentiate into DA neurons after transplantation in a PD model, MSNs after transplantation in a HD model, cholinergic motor neurons after transplantation in an ALS model, and oligodendrocytes after transplantation in a MS model. In acute neurodegeneration, NSCs can be induced to differentiate into cortical glutaminergic neurons or oligodendrocytes after transplantation in ischemic stroke models. After transplantation, pretreated NSCs can mature in the host, stably express specific phenotypes, and integrate into neural circuits to improve the symptoms of ND models. ALS Amyotrophic lateral sclerosis, CNS central nervous system, DA dopamine, ESC embryonic stem cell, HD Huntington’s disease, IPSC induced pluripotent stem cell, MS multiple sclerosis, MSN medium spiny γ-aminobutyric acid–mediated neurons, ND neurodegenerative disease, NSC neural stem cell, PD Parkinson’s disease.

To overcome the limitations of direct transplanted NSCs and given the pathological features of loss of a specific cell type in some NDs, great efforts have been devoted to explore the feasibility of manipulation of NSCs fate prior to transplantation to control the terminal lineage so as to replace lost cells in NDs [31, 32]. Currently, by using chemical-defined systems or ectopic overexpression of critical lineage-specific transcription factors, NSCs from different sources can be directed to differentiate into a specific type of neural lineage cells in vitro, such as DAergic neurons, GABAergic neurons, cholinergic motor neurons, oligodendrocytes, glutamatergic neurons. And subsequent studies have performed in vivo transplantation of predifferentiated cells to investigate their therapeutic role in neurological diseases (Fig. 1).

In this review, we summarized strategies for inducing differentiation of NSCs from different sources in vitro. Then, we outlined the functional improvements and underlying mechanisms of the transplanted preconditioned NSCs in PD and ischemic stroke models. What’s more, we also discussed the limitations of the directional induction of NSCs for clinical translation in NDs and ischemic stroke.

## The directional differentiation of NSCs from different sources in vitro

**Fig. 2:**
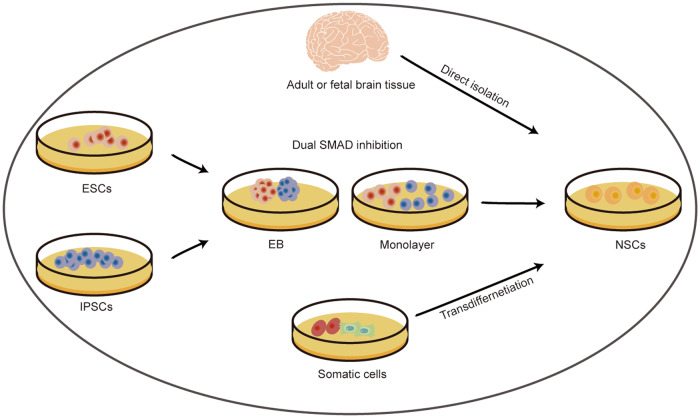
Sources of neural stem cells. Currently, NSCs can be obtained from three ways: 1) isolate from primary CNS tissues, mainly including adult and fetal brain tissue; 2) differentiate from pluripotent stem cells (iPSCs and ESCs) via EB formation or monolayer culture, dual SMAD inhibition can boost the neural induction process; 3) transdifferentiate from somatic cells, such as blood cells and fibroblasts. CNS central nervous system, EB embryoid body, ESC embryonic stem cell, IPSC induced pluripotent stem cell.

At present, NSCs can be derived in three different ways: direct extraction from primary CNS tissues, differentiation of PSCs and transdifferentiation from somatic cells [33] (Fig. 2). NSCs are present throughout the developing brain. And in the adult mammalian brain, NSCs can be found in the subgranular zone of hippocampus, the subventricular zone, and even multiple sites along the entire ventricular system [16, 34, 35]. PSCs, including embryonic stem cells (ESCs) and induced pluripotent stem cells (iPSCs), can be induced to differentiate into NSCs in vitro via two main methods: embryoid body (EB) formation and adherent monolayer culture [36, 37]. Specifically, the process of neural differentiation of PSCs is multistep, first triggering differentiation toward all the three embryonic germ layers by removing mediators that promote self-renewal, and subsequently inhibiting extraembryonic and meso-endoderm differentiation and favoring neural differentiation by culturing the cells in serum-free medium [38]. In addition, dual SMAD inhibition, which simultaneously inhibits transforming growth factor β and BMP signaling pathways, can reduce cultural variability and improve the efficiency of neural induction [38, 39]. Intriguingly, both of these neural induction methods of human PSCs closely resemble the neural induction processes in vivo, giving rise to NSCs with dorsal forebrain identity [38–40]. In addition, neural induction of PSCs can also be achieved by coculture with stromal cell feeder layers, which can provide clues to restrict the fate of PSCs towards neural lineage [41–43]. Induced NSCs can be directly reprogrammed from somatic cells, such as peripheral blood mononuclear cells (PBMNCs), fibroblasts and other cell types [44, 45]. NSCs from different sources can be induced to differentiate into desired neural lineage cells.

**Fig. 3:**
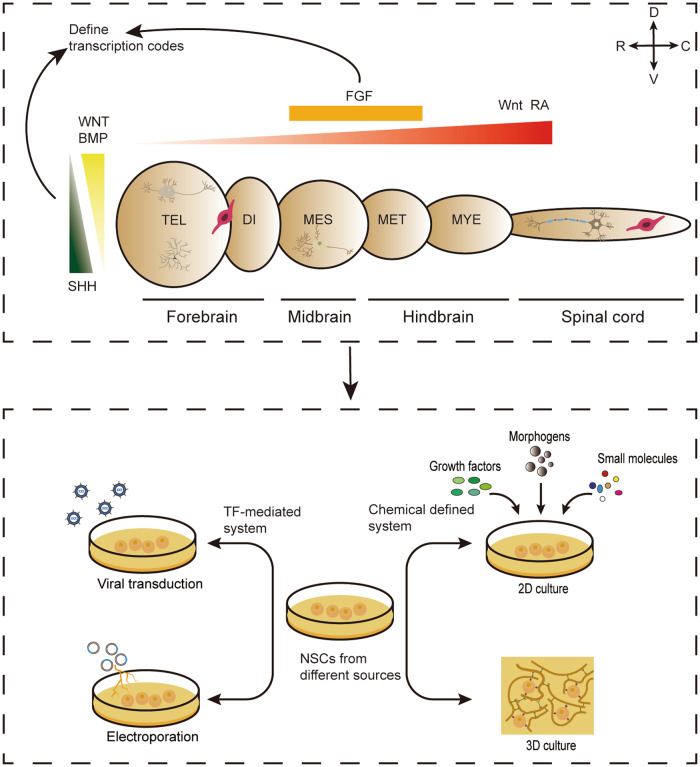
Neurodevelopmental principle for neural lineage subtype specification that guide the directional differentiation of NSCs from different sources in vitro. **A** Morphogen gradients, including BMP, WNT, FGF, SHH and RA, define transcription codes of various neural lineage subtypes in corresponding brain regions during early neural development both along the rostral-caudal and dorsal-ventral axes. The depicted neural lineage subtypes include the MSN in ventral TEL, the cortical glutaminergic neuron in dorsal TEL, the DAergic in ventral MES, the motor neuron in spinal cord, the OPC in forebrain and spinal cord. **B** By using the same chemical or TF patterning principles as seen in vivo, NSCs from different sources can be directional differentiated towards neural lineage subtypes in vitro. The methods of inducing differentiation of NSCs in vitro mainly include external chemical defined system and TF-mediated system. Changes in culture environment include two-dimensional and three-dimensional culture. Overexpression of TFs through viral transduction or non-viral mediated transfection, such as electroporation. BMP bone morphogenic protein, DI diencephalon, FGF fibroblast growth factor, MES mesencephalon, MET metencephalon, MSN medium spiny γ-aminobutyric acid–mediated neurons, MYE myelencephalon, NSC neural stem cell, OPC oligodendrocyte precursor cell, RA retinoic acid, SHH sonic hedgehog, TEL telencephalon, TF transcription factor, WNT wingless-type MMTV integration site family, 2D two-dimensional, 3D three-dimensional.

Understanding the natural development of the nervous system is paramount to manipulating the targeted differentiation of NSCs. The embryonic neural tube undergoes a precise patterning process along the dorso-ventral and antero-posterior axes, resulting in the generation of specific neuronal and glial cell subtypes from NSCs. These intricate developmental events are predominantly orchestrated by organizers, i.e., small groups of cells that release patterning molecules to regulate the fate of NSCs small groups of cells that release patterning molecules to regulate cell fate [38, 46]. Patterning molecules involved in the antero-posterior patterning include fibroblast growth factors (FGF), wingless-type MMTV integration site family (WNT), and retinoic acid (RA), while those affecting dorso-ventral mode include WNTs, bone morphogenic proteins (BMPs), and sonic hedgehog (SHH) [38]. The gradients of morphogens can regulate the intrinsic signaling pathways that define transcription codes [38, 47]. Consequently, it is possible to induce differentiation of NSCs from different sources in vitro by mimicking the regional patterning principles of neural development in vivo, So far, two main approaches of induced differentiation have been developed: chemically defined system and intrinsic transcription factor-mediated method, by which the desired neural lineage cell types can be generated, such as DAergic neurons, MSNs, cholinergic motor neurons, oligodendrocytes, and cortical glutaminergic neurons (Fig. 3). Here, we provide an overview on the progress that has been made in generating several neuronal subtypes as well as oligodendrocytes from different sources of NSCs in vitro (Table 1).

**Table 1:** In vitro differentiation protocols for per neural lineage phenotype and their application in models of neurological diseases.

Phenotypes	Source of NSCs	Differentiation Protocol	Differentiation factors	Phenotypic markers (% cells) in vitro/vivo	Models	Functional outcome	Reference
DAergic neurons	Human fetal VM tissue	Chemical-defined system	BDNF, AA, low oxygen	40–50% MAP2+ 15% TH+/MAP2+	NA	NA	[55]
				NA			
DAergic neurons	Human fetal VM tissue (passage 2)	Chemical-defined system	WNT5 (SHH, FGF8, FGF2 for proliferation)	35%TH+	NA	NA	[62]
				NA			
DAergic neurons	Rat embryonic VM tissue	Transfected by electroporation	Nurr1, Brn4	NA	6-OHDA PD rats	Increased DA level; Improved rotational behavior	[67]
				18%TH+ 14%DAT+			
DAergic neurons	Rat embryonic VM tissue	Transfected by lentivirus	TH, Brn4	65.71 ± 5.18%TH+ 32.28 ± 4.39% DAT+	NA	NA	[66]
				NA			
DAergic neurons	Rat embryonic VM tissue	Chemical-defined system and transfected by lipofectamine	SHH, FGF8 and Wnt5a	a 20-fold TH+ cells increase	6-OHDA PD mice	Increased DA level, improved rotational behavior	[60]
				9.5% TH+			
DAergic neurons	Rodents embryonic cortical tissue	Transfected by retroviruses	Foxa2, Nurr1	37.1% TH+ 55.1% PITX3+/TH+ >78% VMAT2+/TH+	6-OHDA PD rats	Exhibited a mature midbrain DAergic neuronal morphology, improved rotational behavior	[73]
				about 14-fold TH+ cells increase			
DAergic neurons	Rats embryonic cortical tissue	Transfected by retroviruses with appropriate vectors and promoters	Foxa2, Nurr1, ca-PKA	60% TH+/TUJ1+ 80–90% PITX3+/TH+ VMAT2+/TH+ DAT+/TH+	6-OHDA PD rats	Exhibited an extremely mature midbrain DAergic neuronal morphology, no rotational behavior improvement	[74]
				few TH+ cells <100 cells			
DAergic neurons	Primate ESCs (Co-culture with PA6)	Chemical-defined system	NA	25 ± 6% TUJ1+ 35 ± 6% TH+/TUJ1+	6-OHDA PD mice	NA	[43]
				0.7% TH+			
DAergic neurons	Human ESCs (Co-culture with PA6)	Chemical-defined system	SHH, FGF8	46 ± 8% MAP+ 80 ± 11% TH+/MAP+ 32% TH+	NA	NA	[75]
DAergic neurons	Mouse ESCs (Co-culture with MS5)	Chemical-defined system	SHH, FGF8	50 ± 10% TH+/TUJ1+	6-OHDA PD mice	improved rotational behavior	[42]
				10–20% TH+			
DAergic neurons	Human ESCs (EB)	Chemical-defined system	SHH, FGF8	50–60% TH+/TUJ1+ 31.8 ± 3.1% TH+	NA	NA	[77, 79]
				NA			
DAergic neurons	Human PESCs (EB/Dual SMAD inhibition)	Chemical-defined system	SHH C25II, FGF8, PUR and CHIR99021	60–80%/70-100% TUJ1+ 20–40%/30-40% TH+	MPTP PD primates	Increased DA level, improved rotational behavior	[76]
				5.2–8.1% TH+			
DAergic neurons	Human iPSC (EB)	Chemical-defined system	SHH, FGF8	30 ± 5% TH+ 100% GIRK2+/TH+	6-OHDA PD rats	Improved rotational behavior	[78]
				~2% TH+			
DAergic neurons	Human ESCs/iPSCs (Dual SMAD- inhibition)	Chemical-defined system	CHIR99021, FGF8, PUR and SHH-C25II	±75% TH+ ±50% NURR1+ ±80% FOXA2+ ±60% LMX1A+	6-OHDA PD mice/rats MPTP PD primates	Exhibited excellent DA neuron survival, improved motor deficits.	[84]
				6% TH+ (rats)			
DAergic neurons	Human ESCs (Dual SMAD inhibition with EB)	Chemical-defined system	CHIR99021, SHH- C24II	NA	6-OHDA PD rats	Increased DA level, improved motor deficits, showed similar efficacy and potency to fetal DAergic neurons	[82, 178]
				54.2 ± 2.5% TH+ 81% LMX1A+/FOXA2+			
DAergic neurons	Human/primate ESCs/iPSCs (Dual SMAD- inhibition)	Chemical-defined system	CHIR99021, FGF8b and SHH- C25II	43.6 ± 6.2% TH+ 95.3 ± 2.4% NURR1+/TH+ 96.7 ± 1.8% FOXA2+/TH+ 96.5 ± 2.3% LMX1A+/TH+ 56.3 ± 6.7% GIRK2+/TH+	NA	NA	[83]
DAergic neurons	Human iPSCs (Dual SMAD- inhibition)	Chemical-defined system	CHIR99021, FGF8, and PUR	42 ± 4.4% TH+ 19.9 ± 6.9% NURR1+ 70–75% FOXA2+	6-OHDA PD rats /MPTP PD primates	Improved rotational behavior(rats) increased spontaneous movement, extended dense neurites into the host striatum, increased DA synthesis	[81, 85]
				±17% TH+ ±28%TH+/NEUN+(rats) 33.3 ± 24.4% TH+(primates)			
DAergic neurons	Human ESCs (Dual SMAD- inhibition)	Chemical-defined system	CHIR99021, FGF8b, SHH- C25II and SAG	69% TH+ 84% TH+/TUJ1+ >85% GIRK2+/ TH+	6-OHDA PD mice	Displayed A9 characteristics, restored functionality of the reconstructed nigrostriatal circuit, improved motor deficits.	[179]
				68% TH+/survived			
DAergic neurons	Human iPSC (Dual SMAD- Inhibition with EB) Human iNSC	Chemical-defined system	CHIR 99021, FGF8, PUR, BMP5 and BMP7	30–50% TH+/TUJ1+	NA	NA	[86]
				NA			
DAergic neurons	Human ESCs/iPSC (dual SMAD- Inhibition)	Chemical-defined system (3D)	CHIR99021, FGF8b and PUR	47% TH+	Fischer rats	NA	[87]
				8.12% TH+/transplanted 46.7% FOXA2/ TH+			
DAergic neurons	INSCs reprogrammed from PBMNCs	Chemical-defined system	SAG1, FGF8	57.23% TH+ 62.87% TH+/FOXA2 58.69% TH+/NURR1+ 13.84% TH+ 86.78% FOXA2+/TH+ 91.72% NURR1+/TH+ 98.77% GIRK2+/TH+	6-OHDA PD mice	Improved rotational behavior	[88]
GABAergic neurons	Immortalized striatal human NSC line (STROC05)	Chemical-defined system	PUR	6.3% DARPP-32+ 46% TUJ+ 27%+ MAP2+	NA	NA	[99]
GABAergic neurons	Immortalized striatal human NSC line (ST14A)	Chemical-defined system	RA, KCl	74% GABA+	QA HD rats	maintained neuronal GABAergic phenotype, established pre- and postsynaptic contacts with endogenous striatal cells, improved motor deficits	[100]
GABAergic neurons	Immortalized human NSC line (ReNcell VM)	Chemical-defined system	VPA	68 ± 4% MAP2+ 90% GABA+/MAP2+ 54% CALB1+/MAP2+	NA	NA	[101]
			DKK1, SHH	63 ± 4% MAP2+ 96% GABA+/MAP2+ 84% CALB1+/MAP2+			
GABAergic neurons	Human ESCs (EB)	Chemical-defined system	SHH/PUR	90.2 ± 4.2% GABA+/TUJ1+ 89.7 ± 8.3% DARPP32+/TUJ1+	QA HD mice	Projected to the anterior substantia nigra and potentially form connections with DAergic neurons, improved motor deficits	[102]
				62.8 ± 2.6% GABA+ 58.6 ± 3% DARPP-32+/ GABA+			
GABAergic neurons	Human iPSCs (Co-culture with PA6)	Chemical-defined system	BDNF	34.1 ± 4.5% DLX2 27.0 ± 1.7%DARPP-32+ 19.1 ± 2.1% CALB1+	QA HD rat	Improved motor deficits	[41]
GABAergic neurons	Human ESCs/iPSC (Dual SMAD- Inhibition)	Chemical-defined system	DKK1, SHH-C25II	±51% MAP2+ ±78% GABA+/MAP2+ ±60.3% CTIP2+/MAP2+ ±86% GABA+/CTIP2+/MAP2+ ±53% CALB1+/MAP2+ ±70.6% CTIP2+/CALB1+/MAP2+	QA HD rat	Improved rotational behavior	[103]
GABAergic neurons	Human ESCs (Dual SMAD- Inhibition with EB)	Chemical-defined system	XAV939, SAG	±87% DARPP32+/MAP2+ ±89.5% GABA+/TUJ1+ 80–100% DARPP-32+/GABA+ 80–100% CALB1+/TUJ1+	QA HD mice	Improved motor deficits	[104]
				48.7 ± 2.8% DARPP32+/hN+			
GABAergic neurons	Human ESCs/iPSC (Dual SMAD- Inhibition)	Chemical-defined system (3D)	PUR, DKK1	78%MAP2+ 61% GABA+/MAP2+ 55%DARPP-32+/MAP2+ 70%CTIP2+/MAP2+ 46%CALB1+/MAP2+ 100%CTIP2+/DARPP-32+	R6/2 HD mice	Innervated substantia nigra, improved motor deficits.	[105]
GABAergic neurons	Human ESCs/iPSCs (Dual SMAD- Inhibition)	Chemical-defined system	Activin A	20–50%DARPP-32+	QA HD rats	no motor improvement	[106]
				49 ± 5% DARPP-32+/hN+ 86 ± 4.6%GABA+/hN+ 35 ± 8%CALB1+/hN+			
GABAergic neurons	Human ESCs/iPSCs (Dual SMAD- Inhibition)	Chemical-defined system	IWR1	±6%DARPP-32+/Map2b+ ±6%DARPP-32+/CTIP2+ ±60 %CTIP2+	NA	NA	[107]
				NA			
Cholinergic motor neurons	Human fetal cortical NSCs	Chemical-defined system	FGF2	61% HB9+ 50% H9+/ChAT+	NA	NA	[114]
				NA			
Cholinergic motor neurons	HB1.F3 human NSC line	Chemical-defined system and transfected by vector	Olig2, SHH	NA	SOD1G93A mutant mice	Migrated into ventral horn, and replaced lost host motor neurons, delayed clinical onset and extended life span.	[233]
Cholinergic motor neurons	Mouse ESCs (Co-culture with MS5)	Chemical-defined system	SHH, RA and FGF2	±60%HB9+/TUJ1	NA	NA	[42]
				NA			
Cholinergic motor neurons	Human ESCs, primate ESCs (Co-culture with MS5)	Chemical-defined system	SHH, RA	20% HB9+(human) 43% HB9+(primate)	NA	NA	[116]
				NA			
Cholinergic motor neurons	Human ESCs (EB)	Chemical-defined system	FGF2, RA and SHH	>50% ISL1+/TUJ1+/MAP2+ ±50% HB9+/ISL1/2+ ±21% HB9+	NA	NA	[120]
				NA			
Cholinergic motor neurons	Human iPSCs (EB)	Chemical-defined system	PUR, RA	±60%OLIG2+/SOX3+ ±30%ISL1+/TUJ1+	NA	NA	[118]
				NA			
Cholinergic motor neurons	Human iPSCs (EB)	Chemical-defined system	RA, SHH agonist	20%HB9+ >90%ISL1/2+/HB9+ >50%ChAT+/ISL1/2+/HB9+	NA	NA	[119]
				NA			
Cholinergic motor neurons	Human ESCs and iPSCs (EB)	Chemical-defined system	PUR, RA and SAG,	83 ± 1% TUJ1+ 30 ± 6% ISL1+ 16 ± 5% HB9+ 37 ± 2% ISL1+and HB9+	NA	NA	[124]
				NA			
Cholinergic motor neurons	Human ESCs and iPSCs (Dual SMAD Inhibition with EB)	Chemical-defined system	BIO, PUR and RA	40–50%HB9+	NA	NA	[122]
				NA			
Cholinergic motor neurons	Human ESCs and iPSCs	Chemical-defined system (Dual SMAD inhibition)	SAG, RA and CHIR99021	74% HB9+/ISL1+	NA	NA	[125]
				NA			
Cholinergic motor neurons	Human iPSCs (Dual SMAD inhibition)	Chemical-defined system	CHIR99021, PUR and RA	90 ± 9% MNX1 + 95 ± 3% ISL1+ 91 ± 6%ChAT+/MAP2+	NA	NA	[126]
				NA			
Cholinergic motor neurons	Human iPSCs (Dual SMAD inhibition)	Transfected by lentivirus	NGN2, ISL1, LHX3	88.2 ± 3.5% HB9+ 86.5 ± 4.1%ChAT+	NA	NA	[121]
				NA			
Cholinergic motor neurons	Human iNSCs (Reprogrammed from PBMNCs)	Chemical-defined system	RA, SAG1	14.80 ± 0.90% HB9+ 14.40 ± 1.29% ISL1+	NA	NA	[130]
				NA			
Cholinergic motor neurons	Rat iNSCs (Reprogrammed from astrocytes)	Chemical-defined system	RA, SHH	34.1% ± 2.9% HB9+	NA	NA	[131]
				NA			
oligodendrocytes	Human fetal diencephalic/telencephalic tissue	Chemical-defined system	FGF2, NT3 and PDGF-AA	15–20% O4+ 15–20%GalC+	Lysolecithin MS mice	Showed limited myelinating capacity	[141]
				NA			
oligodendrocytes	Human fetal brain tissue	Chemical-defined system	FGF2, NT3 and PDGF-AA	80.5 ± 2.1%A2B5+ 85.4 ± 3.9%O4+ 90%GalC+	NA	NA	[140]
				NA			
oligodendrocytes	Human ESCs (EB)	Chemical-defined system	RA, SHH, FGF2, NT3, PDGF-AA and IGF1	83.95% PDGFRα+ 91.3%NGN2+	Shiverer MS mice	expressed MBP and formed myelin sheaths around nerve fibers	[135, 142]
				NA			
oligodendrocytes	Human ESCs (EB)	Chemical-defined system	RA, PUR/SAG, FGF2, PDGF-AA, T3, low oxygen	Spinal cord 77 ± 13% NGN2+ 38.5 ± 9.0%O4+ 29.9 ± 5.5%MBP+/O4+ Ventral forebrain 91% ± 7% NGN2+ 43% ± 5% O4+ 29.9 ± 5.5%MBP+/O4+	NA	NA	[143]
				NA			
oligodendrocytes	Human ESCs and iPSCs (Dual SMAD inhibition)	Chemical-defined system	RA, SAG, NT3, PDGF-AA and T3	44–70% O4 +	Shiverer MS mice	Achieved mature oligodendrocyte differentiation and formed dense compact myelin.	[145]
				NA			
oligodendrocytes	Human iPSCs (Dual SMAD inhibition)	Transfected by lentivirus	SOX10, OLIG2, NKX6.2	62.1 ± 9.5%-79.0 ± 14.8% O4 + 30.37 ± 7.87% MBP+/O4 +	Shiverer MS mice	myelinated the forebrain, remyelinated the demyelinated spinal cord	[146]
oligodendrocytes	Human iPSCs (Dual SMAD inhibition)	Transfected by lentivirus	SOX10	50–65% O4 +	Shiverer MS mice	myelinated neurons	[147, 234]
				48.13 ± 4.15%MBP+			
oligodendrocytes	Human ESCs and iPSCs (Dual SMAD inhibition)	Chemical-defined system	XAV939, PUR, PDGFRα, IGF-1, cAMP and T3	35% O4+	NA	NA	[149]
				NA			
oligodendrocytes	Human ESCs	Transfected by lentivirus	SOX10, OLIG2	19.24 ± 3.18% O4+ 81.58 ± 3.94% FOXG1+/O4+			[148]
Cortical glutamatergic neurons	Human ESCs and iPSCs (Monolayer)	Chemical-defined system	Noggin	<65% TUJ1+ ±60% VGLUT1+/TUJ1+ <75% TBR1+/TUJ1+ <72% CTIP2+/TUJ1+ <18% CTIP2+/TBR1+/TUJ1+	NA	NA	[155]
				NA			
Cortical glutamatergic neurons	Human ESCs and iPSCs (Dual SMAD inhibition with monolayer)	Chemical-defined system	FGF2, Vitamin A	22–29% TBR1+ 25–30% CTIP2+ 28–36% BRN2+	NA	NA	[164, 165]
				NA			
Cortical glutamatergic neurons	Human iPSCs (EB)	Chemical-defined system	BMP4, WNT3A and cyclopamine	62.2 ± 2.1% TBR1+ ±80% VGLUT1+/TUJ1+	MCAO rats	Alleviated sensorimotor deficits, differentiated to glutamatergic neurons and form excitatory, glutamatergic synapses	[166, 168, 169]
				2.5 ± 0.3% TBR1+			
Cortical glutamatergic neurons	Human ESCs and iPSCs (EB)	Chemical-defined system (3D)	None	30-40% TBR1+ ±30% CTIP2+ ±10%SATB2	NA	NA	[170]
				NA			

The phenotypes of neural lineages, sources of neural stem cells, differentiation protocols, drivers of differentiation, representative phenotypic markers (in vitro) for evaluating the differentiation efficiency and culture homogeneity, expression of representative phenotypic markers after transplantation into corresponding neurological disease model, and improvement of functional outcomes after transplantation are broadly reviewed.

+ represents the percentage of cells stained positive for a specific marker in the differentiation system (in vitro) or in the transplanted population.

*AA* ascorbic acid, *BDNF* brain derived neurotrophic factor, *BIO* GSK3β inhibitor 6-bromoindirubin-3′-oxime, *BMP5* bone morphogenic protein 5, *BMP7* bone morphogenic protein 7, *BRN2* brain-specific homeobox/POU domain protein 2 (POU3F2), *Brn4* brain-specific homeobox/POU domain protein 4, *CALB1* calbindin 1, *Ca-PKA* constitutively active protein kinase A, *CHAT* choline acetyltransferase, *CHIR99021* GSK3β inhibitor, *CTIP2* b-cell CLL/lymphoma 11b(BCL11B)/COUP-TF-interacting protein 2 (COUP-TFII), *3D* three-dimensional, *DA* dopamine, *DARPP-32* dopamine and cAMP-regulated neuronal phosphoprotein 32, *DAT* dopamine transporter, *DKK1* dickkopf-1, *DLX2* distal-less homeobox 2, *ESCs* embryonic stem cells, *EB* embryoid body, *EGF* epidermal growth factor, *FGF2* fibroblast growth factor 2/basic fibroblast growth factor (bFGF), *FGF8* fibroblast growth factor 8, *FGF8b* fibroblast growth factor 8 isoform b, *FOXA2* forkhead box protein A2, *FOXG1* forkhead box protein G1, *GABA* γ-aminobutyric acid, *GalC* Galactocerebrosides, *GIRK2* G protein-activated inward rectifier potassium channel 2 (KCNJ6), *HB9* homeobox HB9/motor neuron and pancrease homeobox 1 (MNX1), *HD* Huntington’s disease, *hN* human nucleus, *IGF-1* insulin-like growth factor 1, *iNSC* induced neural stem cells, *iPSCs* induced pluripotent stem cells, *IWR1* a tankyrase/Wnt inhibitor, *ISL1* ISL LIM homeobox 1, *ISL1/2* ISL LIM homeobox 1/2, *LHX3* LIM homeobox 3, *MAP2* microtubule-associated protein 2, *MBP* myelin basic protein, *MPTP* 1-methyl-4-phenyl-1236-tetrahydropyridine, *MS* multiple sclerosis, *MS-5* stromal cell line derived from irradiated murine bone marrow cultures, *NGN2* neurogenin 2, *NKX6-2* NK6 homeobox 2, *NSCs* neural stem cells, *NURR1* nuclear receptor related 1 protein, *NT3* neurotrophin-3, *6-OHDA* 6-hydroxydopamine, *OLIG2* oligodendrocyte transcription factor 2, *PA6* stromal cell line derived from newborn calvaria tissue of the C57BL/6 mice, *PBMNCs* peripheral blood mononuclear cells, *PD* Parkinson’s disease, *PGDF-AA* platelet-derived growth factor AA, *PGDFα* platelet-derived growth factor -alpha receptor, *PUR* purmorphamine, *PITX3* paired-like homeodomain 3, *QA* quinolinic acid, *RA* retinoic acid, *SAG* smoothened agonist, *SATB2* special AT-rich sequence-binding protein 2, *SHH* sonic hedgehog, *SHH-C24II* recombinant human SHH, *SHH-C25II* recombinant mouse SHH, *SMAD* transcription factor and member of the BMP and TGF-β signaling pathways, *T3* triiodothyronine, *TBR1* T-box brain 1, *SOX3* SRY box 3, *SOX10* SRY box 10, *TH* tyrosine hydroxylase, *TUJ1* neuron-specific class III beta-tubulin (TUBB3), *VGLUT* vesicular glutamate transporter, *VM* ventral midbrain, *VPA* valproic acid, *VMAT2* vesicular monoamine transporter 2, *WNT5* wingless-type MMTV integration site family 5, *WNT5a* wingless-type MMTV integration site family 5a, *XAV939* WNT/β-catenin inhibitor.

### Induction of DAergic neurons from NSCs

Differentiation protocols for DAergic neurons, particularly those targeting midbrain DAergic neurons, have garnered considerable interest in the field of regenerative medicine, owing to their potential to treat PD. Midbrain DAergic neurons are thought to originate from mesencephalic floor plate in embryonic development [48, 49]. The correct establishment of midbrain DAergic precursor domains and the subsequent terminal differentiation of ventral midbrain (VM) DAergic neurons are partly attributed to the synergistic action of regulatory networks controlled by SHH, WNT and FGF [50–52]. In more detail, WNT1 represses the transcription factor Nkx2.2 via the upregulation of Otx2 and the WNT1-Lmx1a autoregulatory loop induces the expression of Lmx1a thus repressing Nkx6-1, both of which promotes the establishment of the midbrain DAergic progenitor domain from ventral mesencephalic NSCs. In addition, the two autoregulatory loop (WNT1-Lmx1a and SHH-Foxa2) induce downstream targets, Pitx3 and Nurr1, which are important factors in the terminal differentiation/survival of midbrain DAergic neurons [51, 53]. FGF8 also provides positional information for the development of midbrain DA neurons [51].

#### Induction of DAergic neurons from NSCs derived from primary CNS tissues

Initially, NSCs were extracted from embryonic or adult brain tissue for targeted differentiation of DAergic neurons. NSCs emanating from the mouse or human VM have been shown to naturally develop into DAergic neurons in vitro [54], and the addition of neurotrophins, such as brain derived neurotrophic factor (BDNF) and glial cell-line derived neurotrophic factor (GDNF) [55, 56], cyclic adenosine monophosphate [57], BMP2 [58], or cytokines [56] has been demonstrated to facilitated the yield of DAergic neurons. In addition, mitogenic factors, such as FGF2 or epidermal growth factor can amplify NSCs to increase the initial number of NSCs used for differentiation [54, 59]. Unfortunately, it has long been reported that the number of rodents VM-derived NSCs differentiated into DAergic neurons decreased after subculture [60, 61], but unlike their rodent counterparts, human VM tissue exhibits a greater ability to expand and differentiate into DAergic neurons [62]. To overcome the reduced ability of dopamine differentiation after passages, modifications of culture conditions such as lowering oxygen levels to mimic the hypoxic conditions of brain development, or the addition of ascorbic acid (AA) has been shown to be useful measures to increase the differentiation of human DAergic neurons after passages [63, 64]. A study adjusted the culture conditions of long-term expanded human VM NSCs, and increased the generation of tyrosine hydroxylase (TH)-positive cells by around 40 times (7% of total cell) through the combined application of BDNF, AA, low oxygen, and prolonged differentiation time [55]. Furthermore, another study revealed that by applying midbrain-specific instructive signals, SHH, FGF8, and FGF2 to proliferating human VM NSCs, these cells maintained the ability to generating midbrain DAergic neurons and extended differentiation in the presence of WNT5 [62]. In addition to manipulation in external culture conditions, the expression of internal key transcription factors can also be regulated to promote dopamine neuronal production from VM NSCs [65], for example, NSCs were transfected with Nurr1/TH and Brn4 by electroporation or lentivirus [66, 67]. And one of the first approaches to boost the yield of DAergic neurons from VM NSCs was based on the both external and internal manipulation [60]. Several studies have revealed that DAergic neuron-inducing activity is specific to VM derived NSCs [68]. Compared with VM NSCs, NSCs from other brain regions seem to be hardly to differentiate into functional DA neurons and lack the ability to release DA [69, 70], suggesting that VM NSCs and non-midbrain NSCs differ significantly in their responses to dopamine-induced signals, possibly due to non-midbrain NSCs lacking appropriate “priming” epigenetic states [71]. Lee et al. demonstrated that by co-expression of Nurr1 and Foxa2 via retrovirus transfection, non-midbrain NSCs gave rise to midbrain DA neuron phenotypes at late stages of midbrain development [72, 73]. The Nurr1+Foxa2 project has been modified in cortical-derived NSCs to mimic the physiological expression pattern of developmental factors of VM NSCs via selecting appropriate vectors and promoters, thus inducing the generation of completely mature midbrain DAergic neurons [74]. Despite extensive efforts, the quest for efficient differentiation of DAergic neurons from tissue-derived NSCs remains elusive.

#### Induction of DAergic neurons from NSCs derived from PSCs

In contrast to the CNS-derived NSCs, the targeted differentiation of PSCs derived NSCs has progressed rapidly, especially the generation of DAergic neurons. It was initially reported that co-cultured with PA6 or MS-5 feeder cells, mouse or primate ESCs can be induced to differentiate into NSCs and further TH-expressing neurons effectively [42, 43]. Furthermore, SHH and FGF8 can provide lineage-specific instructions to enhance the generation of DAergic neurons [42, 75]. Gradually, studies have shown that based on the EBs formation coupled with the action of SHH and FGF8, TH-positive DAergic neurons can be induced successfully from human PSCs [76–79]. With a better understanding of the pattern molecules and transcriptional networks involved in the generation of DAergic neurons in the midbrain during embryonic development, the differentiation protocol has been modified over time. The activation of WNT signaling involved in early caudalization of the cells in the neural plate, was mimicked by the glycogen synthase kinase 3β inhibitor CHIR99021 in vitro, which resulted in an improved midbrain specification reliably and efficiently [76, 80–85]. Compared with DAergic differentiation via a neural rosette intermediate (i.e., the differentiation protocol using SHH and FGF8 only), the DAergic neurons generated from the floor plate were more efficient, both in number and midbrain markers, that is, the number of cells co-expressing TH and fox2 accounted for about 75% of the culture [84]. It is well known that the BMP/SMAD inhibition is used to promote the neural induction of PSCs, but more recently BMP activation has been found to be helpful in the specification of DAergic neurons [86]. A study has revealed that in vitro application of BMP5/7 during the maturation phase can effectively promote the generation of VM DA neurons [86]. Furthermore, the culture system has expanded from two dimensional (2D) to three dimensional (3D) platforms, where cells can be embedded in biomaterials for 3D culture. Schaffer and colleagues have shown that in a 3D thermoresponsive biomaterial platform, by applying the same small molecules used to induce differentiation as in the 2D system, a higher number of TH-positive neurons (~40%) could be generated rapidly after 25 days of differentiation than in their 2D culture control (20%) [87] or other 2D culture systems (15–30%) [84], and these cells exhibited temporal marker expression profiles that resemble natural VM DAergic development [87].

#### Induction of DAergic neurons from NSCs derived from somatic transdifferentiation

DAergic differentiation of induced NSCs (iNSCs) is infancy and there are not as many DAergic differentiation protocols as the other two types of NSCs s. Induced NSCs from PBMNCs can be induced into mature DAergic neurons through a two-stage method. The first stage mediated the generation of DA progenitors mainly through FGF8 and SAG1, a SHH pathway agonist, and the second stage promoted the maturation of DA neurons through a combination of BDNF, GDNF, TGF-β3, AA and other soluble factors [44, 88, 89] . At the end of differentiation, about 60% of cultured cells co-expressed Foxa2 and TH. Moreover, unlike the characteristics of tissue derived NSCs, induced NSCs retained their dopamine differentiation ability after multiple passages [88]. In addition to NSCs derived from PBMNCs, NSCs transdifferentiated from fibroblasts have also been used for DAergic neuron induction, and the DAergic induction protocols of both were similar. The difference is that the addition of BMP5/7 in the maturity phase further increased the generation of DAergic neurons [86].

### Induction of MSNs from NSCs

MSNs in the striatum are the most affected cell type in HD, making them the most suitable target cell type for cell replacement therapy [90]. During embryonic development, the lateral ganglionic eminence (LGE) of the ventral telencephalon gives birth to the MSNs [91]. The concentration gradient of SHH, WNT and BMP can affect the pattern of the dorsal ventral axis, that is, SHH promotes ventral localization of the neural tube and WNT and BMP promote dorsal localization [46]. And the LGE specification is patterned under the impact of antagonistic morphogen gradients. In detail, the repression of WNT together with SHH can efficiently induce the ventral fate of the telencephalic precursors. Furthermore, based on the expression of activin receptors and phosphorylated SMAD2 (an activin signaling pathway component) in the developing LGE, it is speculated that the activation of TGF-β pathway is also involved in the generation of striatum MSN [92]. DLX2 and GSX2 are markers of LGE [93], and MSNs are further characterized by expressed dopamine and cAMP-regulated neuronal phosphoprotein 32 (DARPP-32) and a range of other subtype-specific markers [94, 95]. So far, the generation of MSNs has been primary induced from NSCs from the first two sources, and there is no induction protocol for obtaining MSNs from iNSCs.

#### Induction of MSNs from NSCs derived from primary CNS tissues

NSCs isolated from fetal ganglion eminences can generate up to 25% of DARPP-32 positive neurons in vitro [96, 97], but similar to midbrain DAergic differentiation, this characteristic declines with culture time [98]. Possibly because of previous exposure to a favorable microenvironment, primary tissue-derived NSCs can be predisposed to adopt a specific phenotype, and these predispositions may be largely lost or offset by in vitro cell expansion. Therefore, it may be necessary to provide external cues to support or even increase the MSN differentiation ability of NSCs after passage, such as morphogens [99] and growth factors [97, 99]. A study explored the MSN differentiation conditions of immortalized striatal human NSC line. They compared the chemical induction systems of SHH, SHH/dickkopf-1 (DKK1)/BDNF, Dibutyryl cAMP/valproic acid (VPA)/BDNF, RA, and Purmorphamine, and found that the hedgehog agonist Purmorphamine most remarkably increased the MSN differentiation of NSCs, doubling the number of MSN in the short-term differentiation and tripling the number of MSN in the long-term differentiation [99]. In addition, sequential RA treatment and KCl depolarization can effectively yield 74% functional GABAergic neurons from the immortalized striatum NSCs [100]. Recently, another study found that striatal GABAergic neurons could be reliably induced from immortalized VM NSCs under hypoxic culturing conditions using two- or three-step differentiation protocol based on VPA or SHH and DKK1, respectively [101]. A majority of cultured cells expressed MSN markers and functional glutamate receptors, in addition to releasing GABA on stimulation [101].

#### Induction of MSNs from NSCs derived from PSCs

It is acknowledged that neural induction via EB formation and subsequent exposure to SHH could drive ventral telencephalic fate in human ESCs. Further exploration found that medium dosage of SHH can pattern NSCs into LGE-like progenitor cells, which generate predominantly DARPP32-expressing GABA neurons, ~75% of the total number of cells in culture [102]. Jeon and colleagues induced the generation of NSCs by co-culturing ESCs and iPSCs derived from a patient with juvenile HD with PA6 stromal cells and subsequently producing 27% of DARPP-32 neurons in the presence of BDNF. Additionally, DARPP-32 can be co-localized with LGE markers DLX2 and GSX2, indicating successful generation of the MSN-like cells [41]. In another protocol, neural induction of human ESC and iPSCs was achieved via dual SMAD inhibition, followed by exposure to SHH and WNT inhibitor DKK1, which patterned NSC towards LGE progenitors [103]. A recent study has optimized this protocol by using small molecules to replace protein components, using a chemical cocktail to quickly and efficiently generate GABAergic MSNs from human ESCs [104]. Similar to the improved differentiation protocol of DAergic neurons, a study used a 3D culture method for neural induction and neural specification of ESCs, then matured on 2D laminin-coated plates. The MSNs generated by this 3D-2D method showed electrophysiological activity compared with those generated by 2D method [105]. Interestingly, the addition of Activin rather than SHH also induced the LGE-like progenitor fate after neural induction via dual SMAD inhibition, and the data showed that activin-mediated LGE fate was independent of SHH signaling [106]. Furthermore, another differentiation protocol did not apply SHH or Activin to induce the LGE-like progenitor fate, but continued to inhibit the BMP and WNT signaling pathways via dual SMAD inhibition and the use of IWR1 to regionalize NSCs after neural induction in ESCs [107].

### Induction of cholinergic motor neurons from NSCs

Motor neurons can generally be divided into two categories, depending on the location of the cell body: (I) Upper motor neurons that are located in the cerebral cortex, and (II) lower motor neurons that exist in the brainstem and spinal cord [108, 109]. The differences between the two types of motor cells are not limited to their location, but also manifest in neurotransmitters, targeting, and characteristics upon lesion (reviewed in refs. [108, 109]). Spinal MNs are patterned in the highly restricted foci of ventral neural tube in response to morphogens RA, FGFs, and SHH [110]. In more detail, caudalization of the neural tube is primarily facilitated by RA, produced via the activity of retinaldehyde dehydrogenase 2 [111]. And SHH allows specification of ventral part in the neural tube [112]. The temporal and spatial action of these extrinsic morphogens induce the upregulation of the basic helio-loop-helix (bHLH) transcription factor Olig2, which together with another bHLH transcription factor neurogenin 2 directs the expression of MN fate determining genes such as Islet1 and Hb9 [109, 110]. ALS and other motor neuron diseases characterized by motor neuron injury often result in muscle wasting and even paralysis, and the desire to protect and eventually regenerate motor circuits has prompted attempts to generate motor neurons for translational applications [112]. Here we focus our attention exclusively on the induction protocols for NSCs-derived spinal motor neurons, namely cholinergic motor neurons.

#### Induction of cholinergic motor neurons from NSCs derived from primary CNS tissues

To date, there has been little exploration of spinal motor neurons differentiation protocols from CNS tissue-derived NSCs. FGF2 is well recognized as a mitogen in the CNS, but it has been shown that FGF2 can direct the differentiation of NSCs into spinal motor neurons [113, 114]. In induction medium supplemented with FGF2, about 60% of human fetal forebrain-derived NSCs differentiated into H9 immunopositive cells on day 10, which supports the dual functions of FGF2, i.e., at high concentrations FGF2 primarily serves as a mitogen for NSCs, while at low concentrations it promotes neurogenesis [114]. Furthermore, immortalized telencephalic NSCs transduced Olig2 via retroviral vector expressed motor neuron-specific phenotypes following treatment with SHH, such as Hb9, Islet1 and choline acetyltransferase [115].

#### Induction of cholinergic motor neurons from NSCs derived from PSCs

Compared with CNS tissue-derived NSCs, the motor neuron differentiation protocol of PSC derived NSCs has been widely investigated. Initial protocols for the differentiation of functional cholinergic motor neurons from ESCs have also heavily relied on the use of stromal feeder cells [42, 116]. Furthermore, studies have differentiated NSCs from PSCs via EB formation, followed by treatment with RA and SHH to induce cholinergic motor neuron generation successfully [117–120]. Gradually, most studies have used dual SMAD inhibition or combined with EB formation to accelerate the neuralization of PSCs [121–124]. In addition, some studies optimized ventral and caudal signaling molecules to promote the induction efficiency of motor neurons, for example, Maury et al. activated WNT signal via exposure to appropriate concentration of CHIR to cooperate with RA in caudal optimization, resulting in 80% of cells expressing the MN progenitor cell marker Olig2 [125]. Similarly, a single concentration of ventral morphogen SHH results in a mix of Olig2-expressing motor neuron progenitors with NKX2.2 -expressing interneuron progenitors residing in the adjacent domains. Du et al. used a combination of SHH (induced the Nkx2.2- and Olig2-expressing progenitors) and CHIR (antagonized the induction of Nkx2.2 expression by SHH) to enrich Olig2+/Nkx2.2−MN progenitors, resulting in a purity of more than 90% of motor neurons [126]. Patani et al. described a retinoid-independent protocol for the cadualization of human ESCs based on activin/nodal signaling inhibition, which resulted in the bias to medial motor columnar pools [117]. In addition to exposure to different combinations of patterning molecules that regulate intrinsic transcription factors to induce the generation of motor neurons, some studies have directly transfected transcription factors Neurog2, Islet1, and Lhx3 into human PSCs-derived NSCs via retroviral vectors to promote motor neuron production, which is simple, reliable and efficient [121, 127–129].

#### Induction of cholinergic motor neurons from iNSCs

Currently, few studies have reported cholinergic motor neurons differentiation protocols starting from iNSCs. INSCs can be reprogrammed from astrocytes or PBMCs, and then patterned by RA and SHH in a chemical defined system to confer caudal and ventral anatomical identities, respectively, finally gave rise to Olig2 expressing progenitors. Finally, these progenitors mature into motor neurons under the action of growth factors [130, 131]. But neither protocol was efficient, producing about 15% and 35% HB9-positive cells, respectively [130, 131].

### Induction of oligodendrocytes from NSCs

Oligodendrocytes are glial cells that form myelin sheaths around axons in the CNS, supporting rapid nerve conduction and providing trophic and metabolic support to neuronal cells [132]. During neural development, NSCs give rise to oligodendrocyte precursor cells (OPCs), which are patterned in different regions of the neural tube, such as the ventral and dorsal sides of both spinal cord and forebrain [132–134]. Caudalization of the neural tube is modulated by RA, followed by the generation of Olig2-expressing spinal progenitors in response to ventral signal SHH, which are a source of both motoneurons and OPC [132, 135]. After the generation of motor neurons, Olig2-expressing spinal progenitors downregulate neurogenic transcription factors, and give rise to OPCs that express the oligodendroglial transcription factors Nkx2.2 and Sox10 and the surface markers, such as A2B5, platelet-derived growth factor receptor alpha (PDGFRα) and membrane proteoglycan NG2 [132]. These OPCs differentiate into immature oligodendrocytes expressing marker O4 and further become mature oligodendrocytes expressing myelin marker myelin basic protein [132, 134]. Not only the ventral source, a small number of oligodendrocytes also originate from the dorsal neural tube that is independently of SHH, but Olig2 expression is requisite for dorsal OPC specification [136, 137].

#### Induction of oligodendrocytes from NSCs derived from primary CNS tissues

Genetic modification and culture environmental modification of human NSCs has been tested for the sake of obtaining cell populations enriched in oligodendroglia. One of the first approaches to induce oligodendrocytes from human fetal NSCs was based on the overexpression of the bHLH transcription factor Olig2 via lentiviral vectors. This protocol allowed an increased number of A2B5-positive oligodendroglial precursors in vitro, but fully committed O4-positive oligodendrocytes were not detected after 7 days of differentiation [138]. In a chemically defined system, it has been reported that a combination of FGF2, neurotrophin-3 (NT3) and platelet-derived growth factor-AA (PDGF-AA) successfully increased the proportion of oligodendrocytes expressing O4 and GalCer to 15–20% of the total culture cells from embryonic forebrain-derived NSCs [139]. And another protocol used a similar cocktail combination, resulting in highly pure OPCs from human fetal NSCs. In this study, up to 80–90% of culture cells expressed OPC markers O4, Sox10 A2B5, and PDGF-αR, and about 90% of the cells expressed GalCer with further differentiation [140]. The difference in the efficiency of oligodendrocyte production between the two protocols may be due to the origin of NSCs, as well as the difference in the concentration and duration of action of these factors [139, 140]. In addition, a study committed fetal forebrain-derived NSCs to oligodendrocyte phenotypes by adding PDGF-AA, FGF2, SHH, triiodothyronine, and NT-3, followed by the removal of four factors (PDGF-AA, FGF2, SHH, and NT-3) that promoted the expression of final markers of oligodendrocyte differentiation, with more than half of cultured cells expressing myelin basic protein [141].

#### Induction of oligodendrocytes from NSCs derived from derived from PSCs

Thus far, there have been many attempts to generate oligodendrocytes from human PSCs-derived NSCs. One of the first protocols to induce oligodendrocytes from human ESCs was based on the EBs formation in combination with the activation of RA, SHH and FGF2 signaling [133, 142]. Specifically, ESCs-derived NSCs were patterned to progenitor cells expressing Olig2 and Nkx2.2 in the presence of RA and SHH, and subsequent treatment with FGF2 can inhibit motor neuron differentiation to increase pre-OPCs during the neurogenic phase. Finally, the removal of FGF2 and the addition of PDGF-AA, insulin growth factor 1 and NT3 promotes the transition of pre-OPCs to OPCs, and ~80% of cultured cells expressed OPC markers, such as PDGFRα and NG2 [133, 135, 142]. But unfortunately, this induction protocol takes a long time, at least 3 months, to generate OPCs from human PSCs [133, 142]. To address the limitation of long differentiation time, some groups have modified the induction protocol. Franklin et al developed a physiological oxygen tension protocol to generate oligodendrocytes from human ESCs under low oxygen conditions, mimicking the environment of the developing brain [143]. And the results indicated that hypoxic conditions could not only accelerate the overall differentiation process, but also significantly improve oligodendrocyte production [143]. Fossati and colleagues induced the generation of NSCs by using dual SMAD inhibition rapidly and modified the previous oligodendrocyte differentiation conditions slightly, thus speeding up the timetable of glial induction and resulting in most of cultured cells displaying the late OPC marker O4 [144, 145]. However, these optimized protocols shorten the differentiation cycle to 70 days at most, and primary rate-limiting steps are oligodendroglial specification and differentiation, some studies have accelerated the generation of oligodendrocytes by overexpression of transcription factors. More recently, an effective strategy that facilitated the generation of O4- expressing oligodendrocytes to 70% within 28 days of differentiation by using a combination of three transcription factors, Olig2, Sox10, and Nkx6.2 has been reported [146]. Verfaillie and colleagues described that overexpression of a single transcription factor, Sox10, was sufficient to generate similar levels of O4+ cells from human PSCs derived NSCs within 22 days [147]. In addition to the generation of spinal cord OPCs and oligodendrocytes via the use of caudal morphogen RA, several RA-independent approaches that favor telencephalic OPC generation have been reported [143, 148, 149]. A study accelerated the production of RA-independent telencephalic oligodendrocytes by enhancing neural induction using dual SMAD inhibition in conjunction with the tankase inhibitor XAV 939 (antagonizing WNT signaling) [149]. More recently, Xiong and colleagues first promoted the generation of ventral forebrain NSCs of ESCs by dual SMAD inhibition and activation SHH signals, and subsequently demonstrated that overexpression of Sox10 and Olig2 in these cells was sufficient to generate forebrain mature oligodendrocytes at day 40 of differentiation [148].

### Induction of cortical glutamatergic neurons from NSCs

Glutamatergic pyramidal neurons are the vast majority of excitatory nerve cells in the cerebral cortex that mediate myriad information processing streams and output channels [150]. In brain development, all cortical glutamatergic neurons originate from the embryonic dorsal telencephalon [151], which is patterned by WNTs and BMPs that are derived from the cortical hem [152, 153]. Early dorsal forebrain primordium co-express Pax6 and Otx1/2, and over time, these cells differentiated into cortical glutamatergic neurons, displaying unipolar and pyramidal morphology, and expressing TBR1, CTIP2, and vesicular glutamate transporters [154, 155]. Glutamatergic neurogenesis is also present in adult neurogenic niches, SVZ and SGZ [156, 157]. Interestingly, the pattern of transcription factor expression during adult glutamatergic neurogenesis is akin to the sequential expression of transcription factors in cortical glutamatergic neurons during the embryonic period, indicating that the genetic program specifying the fate of glutamate is spatially and temporally conserved [156].

#### Induction of cortical glutamatergic neurons from NSCs derived from primary CNS tissues

Existing differentiation protocols tend to generate a mixture of cortical neurons from primary CNS tissue derived NSCs, rather than differentiating specifically into cortical glutamate neurons. NSCs isolated from cortex of human fetuses retained their regional identity and differentiated primarily into cortical GABAergic interneurons and glutamatergic neurons after the removal of the mitogen [158]. In addition, immortal fetal cortical NSCs lines also showed similar differentiation characteristics [159, 160], and a cortical human NSCs line CTX0E16 generated about 40% CTIP2-positive cells with typical pyramidal neuron morphology in vitro [159]. More recently, a study indicated that embryonic mouse dorsal cortical derived NSCs developed towards cortical glutaminergic neurons under FGF at below proliferative concentrations, possibly due to the endogenous and transient wave of BMP signals induced by low FGF2 [161].

#### Induction of cortical glutamatergic neurons from NSCs derived from derived from PSCs

As we mentioned earlier, forebrain identity is the default procedure after neural induction of PSCs, and it has been shown that human PSCs predominantly differentiate into dorsal telencephalic NSCs after neural induction without the need for additional patterning morphogens, which is attributed to endogenous WNT signaling [153, 162]. In addition, several differentiation protocols, involving either EB formation or monolayer culture, enhanced neural induction of human PSCs through the use of the BMP inhibitor Noggin, thereby increasing the yield of cortical glutamate-like neuron production [155, 163], and most of these cells generated here exhibited an identity corresponding to deep layers rather than upper layers, which was determined by the expression of layer-specific markers during the process of differentiation [155]. In contrast, another differentiation protocol described that the equivalent proportions of deep and upper layer neurons can be generated from human PSCs when combined with dual inhibition of SMAD signaling and retinoic acid signaling [164, 165]. Nevertheless, recent studies have shown that the use of cyclopamine (an inhibitor of SHH) can promote the population of cortical glutamate neurons by inhibiting ventral differentiation from iPSCs [166–169]. Compared with 2D induction protocols, the generation and maturation of cortical glutamatergic neurons were further promoted by cultivating human PSCs derived NSCs in the PDMS-based 3D culture system [170].

## Therapeutic potential of induced directed differentiation of NSCs in neurological disease models

As mentioned above, NSCs have been directed to differentiate into specific lineages of cells expressing corresponding transcription factors and markers (Table 1), displaying cellular morphology, as well as manifested by electrophysiological properties in vitro [171, 172]. Furthermore, whether predifferentiated NSCs can survive, stably express the desired cell subtypes, and functionally integrate into the host brain of neurological models are increasingly being investigated (Table 1). The main neurological disease models currently involved include PD, HD, MS, ALS, and ischemic stroke. Here we primarily outlined the therapeutic potential of NSCs-derived specific phenotypes in chronic neurodegenerative disease PD and acute neurodegeneration ischemic stroke.

### Parkinson’s disease

PD is a progressive neurodegenerative disorder characterized pathologically by the degeneration of DAergic neurons in the substantia nigra pars compacta, with a subsequent loss of DAergic axon terminals innervating the striatum, which results in motor disorder [173]. Currently, the main treatment for PD is dopamine replacement therapy. However, it can only relieve the symptoms of PD without delaying the progression of PD [174]. Regarding to the success of targeted differentiation of VM DAergic neurons in vitro, some studies have transplanted these cells into different models of PD to observe their therapeutic effects at the behavioral, cellular, and molecular levels.

Most of the studies mentioned above implanted pre-differentiated NSCs into the striatum of 6-OHDA or MPTP-induced PD models, resulting in sensorimotor improvements, such as increased spontaneous activity and reduced circling behavior [60, 67, 73, 76, 78, 81, 82, 84, 85, 88]. Ectopic implantation is considered given that VM tissue grafts placed in the substantia nigra are unable to extend axons long enough to reach their target area, the striatum, to form complex neural circuits [42, 175–177]. After implantation, some of these preconditioned NSCs could survive in the PD animal model and exhibit a phenotype of nigra DAergic neurons, with increased dopamine levels [60, 67, 73, 76, 81, 82, 84, 85, 178]. The development of experimental techniques over the past decade has led to a refined understanding of how transplanted cells integrate with circuitry of the host nervous system [179]. Previous optogenetic and electrophysiological studies have demonstrated that ectopically transplanted NSCs-derived midbrain DAergic neurons are spontaneously active and receive appropriate presynaptic input from the host [180, 181], and the recent availability of the monosynaptic rabies tracing technique allowed us to further investigate the sources and extent of host synaptic inputs comprehensively [182]. For example, it has been shown that despite the ectopic intrastriatal location of NSCs-derived midbrain DAergic neurons, they can receive excitatory and inhibitory inputs from host cortical, striatal and pallidum neuronal subtypes, which are acknowledged to modulate the function of endogenous DAergic neurons in the substantia nigra, and this host afferent pattern helped to explain the proper regulation of DA release in “ectopic” intrastriatal VM-patterned grafts [183]. But the ectopic location of grafted cells may hinder the maximization of their function due to incompatibility with the physiological anatomy. To achieve a more complete circuit repair, homotopic transplantation to the substantia nigra is gradually being performed in rodent PD models [178, 179, 183]. Using VM DAergic cells derived from green fluorescent protein transgenic mouse embryos, earlier studies demonstrated the anatomical and functional reconstruction of nigrostriatal pathway after homotopic transplantation [175, 184]. Subsequently, it was observed that by using species-specific antibodies or genetic labeling, VM DAergic cells derived from NSCs innervated caudate putamen or prefrontal cortex, the main target of endogenous substantia nigra pars compacta (A9) or ventral tegmental area (A10) VM DAergic neurons, indicating that the grafted human VM DAergic neurons are the mixture of A9 and A10 phenotypes [179, 185, 186]. Interestingly, the innervated areas of intrastriatal VM DAergic neuron grafts were almost identical to those of the intranigral grafts, suggesting that the graft path-finding and target projection were largely determined by the cell-intrinsic factors [179, 183]. Therefore, the present studies showed that both ectopic and homotopic transplanted VM DA neurons can perform presynaptic and postsynaptic integration, and it was natural to assume that the reconstructed functional nigra-striatal circuit resulted in motor recovery in PD models, as demonstrated by the use of optogenetic and chemogenetic tools [179, 180, 187].

In addition to the transplant location, the optimal time window of DAergic neuron differentiation for transplantation is also important for the reconstruction of the nigra-striatal circuit [188, 189]. Using VM tissue as the donor, the maturity of the donor cells at transplantation significantly affected the grafts composition and functional outcomes. Specifically, donor tissue isolated before the peak of DA neurogenesis, embryonic day (E) 12, produced more DAergic neurons in the graft, which was attributed to increased DA neuroblasts survival and proliferation at the time of implantation [190–192]. Further studies on embryonic development showed that different DAergic subtypes originated from different progenitor pools and had different birth dates, with A9 DAergic neurons emerging earlier during DA neurogenesis [193–195], thus grafting of younger ventral midbrain donor tissue (E10) enriched A9 population and enhanced motor recovery [189, 193]. This may also be the reason why some studies have selected younger donor tissues for DAergic differentiation of ventral midbrain derived NSCs, and these pretreated VM NSCs grafts showed a higher TH-positive cell survival rate than fetal VM tissue grafts [60]. Currently, despite advanced protocols for the targeted differentiation of NSCs from two other sources into DAergic progenitor cells suitable for transplantation [42, 76, 78, 82, 84, 85, 88, 178, 179] and their rapid transition to clinical trials [196], the optimal stage of differentiation for transplantation has rarely been explored. On the one hand, the more mature the cells are in vitro, the more fragile they are and the more difficult they are to survive after transplantation. On the other hand, a higher degree of stemness is responsible for a greater chance of survival, but may result in insufficient regional regulation to generate mature DAergic neurons in vivo [188]. Therefore, most studies have focused on transplanting their derived VM progenitor cells at intermediate stages of differentiation, aiming to balance the ability to survive and mature [82, 84, 88, 186, 197]. A recent study attempted to transplant progenitor cells at different times of DAergic differentiation to determine the most appropriate transplantation time window, and it was surprising to find that grafts derived from younger progenitor cells consisted of the highest proportion of VM DAergic neurons and the lowest proportion of non-target cell types, showing intensive innervation ability as well as increased DA levels [188]. Although the donor age effect was observed in both human PSC lines, there was some variability across cell lines, highlighting the significance of characterizing cells in vivo on the basis of different cell lines or standardizing differentiation protocols [188]. Furthermore, in this study, some commonly used mesencephalic floor plate markers were used to determine the optimal differentiation time window of transplanted cells, such as FOXA2 and OTX2 [188]. But by using RNA sequencing, another study found that the high DAergic yield and their functional maturation in vivo positively correlated with a specific group of markers associated with the caudal midbrain, rather than the levels of those commonly used markers. According to these markers, a good manufacturing practice differentiation protocol for VM DAergic progenitor cell production was developed through the use timed delivery of FGF8 and a number of other adjustments [197]. In conclusion, the ability of these markers to more precisely predict graft outcome will accelerate the clinical application of stem cells. In the future, a panel of markers can be refined and identified at the progenitor stage in vitro to predict more functional mature A9 DAergic neurons in vivo.

### Ischemic stroke

Ischemic stroke is a cerebrovascular event that, although not classified as a neurodegenerative disease, also presents pathological cell death in the infarct area, including different types of neurons and glial cells [198]. In recent years, the limited treatments for ischemic stroke include endorvascular surgery (called thrombectomy) or intravenous administration of alteplase (called thrombolysis) for the purpose of restoring blood flow [199]. However, due to the narrow therapeutic window, some contraindications, low efficacy to recanalize the large artery via thrombolysis, and even the reperfusion injury after recanalization, only a small percentage of ischemic patients can benefit from these two treatments [200–203]. Possibly inspired by replacement therapy for specific cells lost in NDs, recently there have been studies targeting different lost cell types in ischemic stroke to transplant specific cells for replacement to reconstruct damaged neural circuits [166, 168, 169].

A clinical and imaging study showed that the distribution of damaged cells under the most severe symptoms in stroke patients was often not in the striatum, suggesting that cell replacement strategies should emphasize reconstructing the damaged cortex rather than the striatum [204]. Therefore, some studies have implanted progenitor cells with a cortical glutaminergic phenotype derived from PSCs into the cortex of rat models of ischemic stroke and found that these cells could alleviate sensorimotor impairment at 2 or 6 months after transplantation [166, 169]. Behavioral improvements at the early time point of 2 months post-transplantation were most likely not attributable to neuronal replacement and circuitry integration. The use of human-specific cytoplasmic markers combined with green protein immunostaining revealed that axonal projection of transplanted cells extended to both ipsilateral and contralateral hemispheres [169]. Further by using rabies virus–based transsynaptic tracing and optogenetics, it was found that the contralateral somatosensory cortex received functional monosynaptic input from the transplanted neurons [166]. In addition to functional efferent integration, transplanted cells also received synaptic inputs from the thalamocortex and were able to modulate their own activity in response to physiological sensory stimuli [168]. The functional circuit reconstruction is responsible for the recovery of motor function at late time points after transplantation [166]. Except for cortical glutamatergic excitatory neurons, another study identified the phenotype of transplanted predifferentiated NSCs as GABAergic inhibitory neurons. NSCs isolated from human fetal SVZ were predifferentiated in the presence of BDNF, and the predifferentiated cells were subsequently transplanted into the striatum and cortex of cerebral ischemic rats. Histopathology 28 days-post transplant indicated that these cells stably expressed the GABA phenotype, increased GABA levels, and exerted their trophic effects to promote endogenous neurogenesis, which may lead to faster functional recovery in cerebral ischemic rats than those treated with undifferentiated NSCs [205]. In addition to sensorimotor dysfunction, most stroke survivors suffer from cognitive impairment, which may be related to demyelination of the brain’s white matter, resulting from oligodendrocyte death [206, 207]. Xu et al. proposed a two-step protocol to derive NG2-positive OPCs stably and rapidly from iPSCs, first inhibiting SHH to generate NPC and then overexpressing Olig2 [208]. After transplanting OPCs into the cerebral ventricles of ischemic rats, it was found that these cells could protect host neurons from death under the ischemic environment by suppressing inflammatory and immune responses. Furthermore, these cells rescued learning and memory loss to some extent by facilitating the remyelination process in ischemic stroke rats [208].

## Discussion and future directions

Based on numerous studies of cell transplantation, it can be inferred that both pre-transplantation history and source of donor cells are critical factors that affect the outcome of transplantation. In terms of pre-transplant history, most of the protocols for targeted differentiation of NSCs from different sources involve external chemical-defined system through patterning cues or intrinsic ectopic overexpression of lineage-specific transcription factors. Currently the external culture system is gradually moving from the initial co-culture or use of poorly defined xenogeneic factors toward a fully chemical-defined, and xeno-free condition, which encourages the establishment of the more robust differentiation protocol [209]. However, most existing targeted differentiation methods tend to generate heterogeneous cultures containing the cell type of interest as well as other undesirable phenotypes. Consequently, further exploration of the use of morphogens, growth factors, and small molecules in concentration, sequence, and duration is warranted. On the other hand, when manipulating gene overexpression, full consideration should be given to their expression patterns during neural development in order to achieve adequate maturation of differentiated cells and long-term phenotypic maintenance [74]. but virus-mediated multigene transduction is somewhat cytotoxic leading to poor implantation of donor cells, and another concern is that viral integration could disrupt normal gene expression, thus may not be suitable for clinical scenarios. In addition to the two approaches to orchestrate the directed induction differentiation of NSCs, increasingly, it has been shown that epigenetic machinery can regulate the interaction between activation and inhibition of various developmental signaling pathways in neural differentiation [210], such as finely tuning genetic programs to coordinate distinct neural lineage differentiation [211]. With the current advances in RNA interference, they could also be used to guide the targeted differentiation of NSCs [212–215]. In addition, Traditional two-dimensional induction methods provide basic soluble regulators to control the fate of NSCs. However, stem cell behaviors are regulated by different physiological, physico-chemical and physico-mechanical cues, so three-dimensional induction involving biological materials is increasingly being emphasized to effectively control the fate of stem cells [216–218]. With the rise of interdisciplinary of medicine and engineering, conducting polymers have been proved to induce the directional differentiation of NSCs through electrical stimulus in vitro [219]. All these efforts are aimed at promoting the targeted differentiation of NSCs and obtaining specific neural lineage cells in vitro.

The selection of primary CNS tissues and cell types for the generation of specific neuronal lineage phenotype also requires to be taken into account, as it may affect the efficiency of neuronal lineage differentiation and the effectiveness of transplantation. The potential of primary CNS tissue-derived NSCs varies depending on the developmental stage at which they are obtained and the site from where they were isolated [220]. For instance, under identical culture conditions, NSCs derived from ventral mesencephalic produced more DAergic neurons than those from striatum [68], and A9 neurons are produced earlier than A10 neurons during neural development [189], so NSCs derived from VM at an early stage is more conducive to the generation of A9 DAergic neurons. In contrast, NSCs derived from neural induction of PSCs tend to be at an earlier stage, and they can actively respond to multiple patterning molecules to differentiate into different neural lineages. However, it is gradually recognized that epigenetic differences exist between different PSC strains, leading to deviations in lineage differentiation or different generation efficiencies of the same lineage [163, 188, 221]. These differences highlight the need to further compare the results of directed differentiation of neural lineages across different PSC lines. In contrast to tissue-derived NSCs or ESCs, iPSCs and iNSCs are not subject to ethical concerns. In particular, patient-specific iPSCs or iNSCs are targeted to differentiate into neural lineage cells that will match individual immunity—a goal long pursed in regenerative medicine, but before they can be reasonably used for cell therapy, it is critical to understand and correct any intrinsic defects in these cells [119]. Neural grafts derived from human iNSCs are less likely to produce fast-growing tumors following grafting compared with grafts of PSCs [222, 223]. Currently, several strategies are actively being taken to address the possibility of potentially pathological growth of grafted cells from various sources, such as the use of cell-sorting techniques to remove Off-target contaminating cell types prior to transplantation [81, 224, 225] or the transduction of ligand-activated suicide genes to ablate proliferating cells in vivo [226, 227]. With the rapid development of induced neuron (iN) technology in recent years, it is possible to directly reprogram somatic cells to obtain functional neurons [93]. However, the iN method converts somatic cells directly into non-dividing neurons rather than fate committed neuronal progenitors, and these non-dividing neurons often tend to survive and integrate poorly in the host brain after transplantation [228].

In addition, the specific pathological states presented by different neurological diseases should be fully considered before donor cell transplantation, which may affect the cell transplantation strategy in a degree. In neurodegenerative disorders, the outcome of cell replacement relies on the complexity and precision of the connection patterns that need to be restored [229]. In the case of Parkinson’s disease, only a partial pattern repair can result in significant functional recovery. Ectopic transplantation of DAergic cells, also known as paracrine strategy, can restore the efficient release of dopamine to regulate the substantia nigrostriatal circuitry [60, 67, 73, 76]. Unlike Parkinson’s disease, for local neuronal degeneration due to HD or ALS and global neurodegeneration due to ischemic stroke, it is necessary to re-establish the specific afferent–efferent connections between the graft and host, emphasizing the importance of homotopic transplantation [229]. So far, based on advances in nanotechnology, molecular biology and imaging techniques, transplanted cell tracing technology has shifted from ex vitro detection to in vivo imaging [230], and further combining with viral or genetic labeling strategies can help researchers explore the synaptic connection between transplanted cells and host brain [179].

In addition to transplantation of directionally induced neural stem cells, in vivo cell reprogramming, the in-situ conversion of glial cells to functional new neurons is also a promising neural regeneration strategy [9]. Furthermore, transplanted exogenous stem cells can be genetically engineered to steadily produce growth factors that support the repair of dysfunctional endogenous neurons [231]. For example, human iPSC-derived neural progenitor cells genetically engineered to stably produce GDNF can delivery GDNF after transplantation to protect degenerated neurons in PD models and ALS models [232]. As a result, researchers have poured a great deal of effort in different ways, towards the same goal: to promote nerve repair or regeneration.

Recent preclinical studies have demonstrated the safety and efficacy of DAergic neurons derived from PSCs. Based on these promising results, clinical trials are being conducted in different countries [76, 224]. PD clinical trials provide important guidelines for other derived neural lineage cells venturing into these uncharted territories, and we believe that neural lineage cells derived from different sources of NSCs, especially PSCs, will be tested in clinical trials in the near future to develop treatments for related neurological diseases.
